# Endometrial progesterone resistance and PCOS

**DOI:** 10.1186/1423-0127-21-2

**Published:** 2014-01-09

**Authors:** Xin Li, Yi Feng, Jin-Fang Lin, Håkan Billig, Ruijin Shao

**Affiliations:** 1 Department of Physiology/Endocrinology, Institute of Neuroscience and Physiology, The Sahlgrenska Academy, University of Gothenburg, Gothenburg 40530, Sweden; 2 Department of Gynecology, Obstetrics and Gynecology Hospital of Fudan University, Shanghai 200011, China; 3 Department of Integrative Medicine and Neurobiology, State Key Lab of Medical Neurobiology, Shanghai Medical College and Institute of Acupuncture Research (WHO Collaborating Center for Traditional Medicine), Institute of Brain Science, Fudan University, Shanghai 200032, China

**Keywords:** Progesterone resistance, Progesterone receptor isoforms, Endometrium, PCOS

## Abstract

Polycystic ovary syndrome (PCOS) is a state of altered steroid hormone production and activity. Chronic estrogen exposure or lack of progesterone due to ovarian dysfunction can result in endometrial hyperplasia and carcinoma. A key contributor to our understanding of progesterone as a critical regulator for normal uterine function has been the elucidation of progesterone receptor (PR) expression, regulation, and signaling pathways. Several human studies indicate that PR-mediated signaling pathways in the nucleus are associated with progesterone resistance in women with PCOS. The aim of this review is to provide an overview of endometrial progesterone resistance in women with PCOS; to present the PR structure, its different isoforms, and their expression in the endometrium; to illustrate the possible regulation of PR and PR-mediated signaling in progesterone resistance in women with PCOS; and to discuss current clinical treatments for atypical endometrial hyperplasia and endometrial carcinoma in women with PCOS and accompanying progesterone resistance.

## Introduction

### Polycystic ovary syndrome: a multifactorial disease

Polycystic ovary syndrome (PCOS) is a heterogeneous hormone-imbalance disorder [1] that occurs in approximately 4% or 18% of reproductive-aged women worldwide [2]. Although PCOS is usually diagnosed during the early reproductive years, the precise etiology and pathogenesis of PCOS remain uncertain. The main clinical complications of this disease include hyperandrogenism, chronic oligo/anovulation, polycystic ovaries, insulin resistance, and type 2 diabetes mellitus [1,3]. Because the signs and symptoms of PCOS can vary among individuals, and because no single factor is likely to explain the constellation of abnormalities in this syndrome, it is often difficult to accurately diagnose women with PCOS. According to the Rotterdam criteria provided by the American Society for Reproductive Medicine and the European Society for Human Reproduction and Embryology [4], at least two of the following diagnostic criteria should be met: oligo/anovulation; clinical signs of hyperandrogenism (e.g., hirsutism and acne) and/or biochemical measurements; or enhanced polycystic ovaries and/or multiple discrete follicles in diameter in one ovary as detected by ultrasonography. In addition, other androgen excess disorders and specific etiologies such as congenital adrenal hyperplasia, Cushing’s syndrome, thyroid hormone abnormalities, hyperprolactinemia, and ovarian/adrenal tumors need to be excluded.

In clinical practice, 75% of women with PCOS suffer from anovulation infertility [5] and 50% of them experience recurrent pregnancy loss [6]. It is, however, not clear whether these defects are caused by uterine dysfunction itself or by the interrupted interaction between uterine cells and the developing embryo. Additionally, the chronic anovulation seen in PCOS implies prolonged estrogen excess or lack of progesterone and results in atypical endometrial hyperplasia, which is the precursor of endometrial carcinoma [7,8]. Indeed, young women with PCOS-induced endometrial hyperplasia are more likely than non-PCOS women to develop endometrial carcinoma [9]. Therefore, it is important to understand the mechanisms and consequences behind the pathophysiological changes in the endometrium in women with PCOS in order to develop effective treatments to prevent the development of endometrial carcinoma.

This review focuses on progesterone activity in the endometrium and the structural characteristics of human progesterone receptor (PR) isoforms. The review further compares PR isoform expression and regulation in women with PCOS in relation to the clinical consequences of progesterone resistance. We also discuss the principal therapeutic treatments that have been used to improve progesterone sensitivity in women with PCOS.

## Review

### Endometrial progesterone resistance

Progesterone is a key steroid hormone produced mainly by the ovaries, and its synthesis and secretion are primarily regulated by luteinizing hormone during the menstrual cycle and by human chorionic gonadotropin during pregnancy [10]. Progesterone is absolutely required for uterine implantation, decidualization, and maintenance of pregnancy [11]. The uterine endometrium includes epithelial cells, stromal cells, immune cells, and blood vessels [12], and both epithelial and stromal cells are exquisitely sensitive to steroid hormone stimulation in women during the menstrual cycle [13]. For example, 17ß-estradiol (E2) drives epithelial cell proliferation whereas progesterone inhibits E2-stimulated epithelial cell proliferation [10].

Progesterone resistance implies a decreased responsiveness of target tissue to bioavailable progesterone [14], and such an impaired progesterone response is seen in the endometrium of women with PCOS [15,16]. Women with PCOS often present with an abnormal menstrual cycle and anovulation, and this results in minimal or absent P4 production as evidenced by the fact that the endometrium is thicker in women with PCOS than in healthy women [17]. In the literature, progesterone resistance generally refers to women who suffer from endometriosis. Endometriosis is an E2-dependent disease but it alters a subset of progesterone-regulated genes and pathways in the endometrium [18]. Although gene expression analysis of PCOS endometrium reveals progesterone resistance and candidate susceptibility genes in women with PCOS [16,19], the molecular mechanisms underlying endometrial progesterone resistance or sensitivity in these patients are not completely understood.

### Current understanding of progesterone receptors

**Figure 1:**
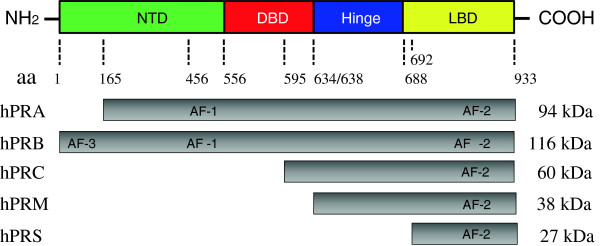
**The general structure of human progesterone receptors.** The PR protein consists of four structurally and functionally distinct domains: the N-terminal transactivation domain (NTD), the DNA binding domain (DBD), a small hinge region, and the C-terminal ligand-binding domain (LBD). The LBD shares the greatest degree of homology among the different human PR isoforms. In addition to hPRA and hPRB, hPRC lacks the first of two zinc fingers and be defective in DNA binding, whereas hPRM lacks both NTD and DBD. The structure of both hPRC and hPRM predicts extra-nuclear localization. hPRS contains only LBD and may be involved in the progesterone-induced nuclear translocation. hPR, human progesterone receptor; aa, amino acids; AF, activation function.

**Figure 2:**
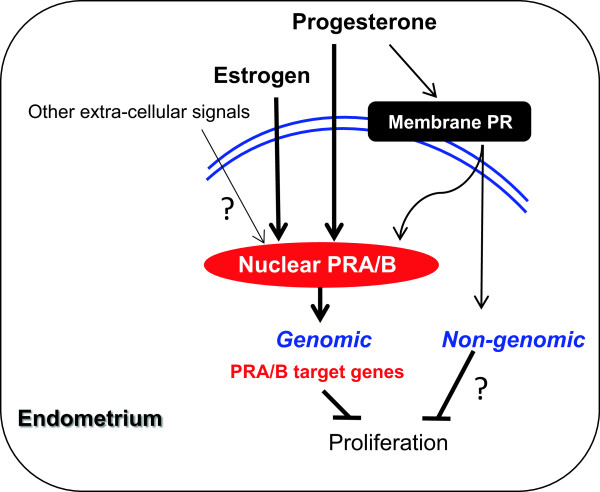
**Progesterone receptors mediate genomic and non-genomic actions in human endometrium.** Uterine responsiveness to progesterone is dependent on nuclear PRA/B. Progesterone also acts through different membrane-bound PRs that mediate non-genomic activities. In addition to progesterone, nuclear PRA/B has been shown to be under estrogenic control in human and animal uteri. Although the non-genomic effects of membrane-bound PRs in the endometrium are unclear, several studies have suggested that the PRA/B-regulated genes are involved in the regulation of endometrial proliferation. PRA/B, progesterone receptor isoforms A and B.

PRs mediate both genomic and non-genomic signaling pathways. Uterine responsiveness to progesterone is dependent on nuclear PR, which is a member of the steroid receptor superfamily and regulates transcription of target genes [11,18]. Like other steroid hormone receptors, PR is composed of a variable N-terminal transactivation domain, a highly conserved DNA-binding domain, a hinge domain, and a ligand-binding domain [20] (Figure 1). In the absence of an activated ligand, PR is inactivated through association with various heat shock proteins (hsp), including hsp90, hsp70, and hsp40, and other co-repressor proteins [10]. Ligand binding causes release of the multiple hsp-subunit complex and the PR undergoes a conformational change that allows the receptor dimer to interact with specific progesterone response elements located within the regulatory regions of its target genes [20] (Figure 2). There are two predominant isoforms of the PR, PRA and PRB [10]. Both are encoded from the same *PR* gene through the use of alternative promoters and translation start sites. PRA lacks the N-terminal 164 aa of PRB [21] (Figure 1). Importantly, the two PR isoforms are not functionally equivalent, and *in vitro* experiments show that PRA functions as a transcriptional inhibitor of PRB when PRA and PRB are co-expressed [21]. *In vivo* knockout studies reveal that mice specifically lacking uterine PRA but not PRB fail to display progesterone-induced inhibition of E2-induced cell proliferation and that this results in uterine dysfunction and infertility [22]. These animal studies suggest that PRA might play a pivotal role in uterine function.

Both PRA and PRB can either homodimerize or heterodimerize *in vivo*[23], and the relative expression of the two isoforms varies dramatically in different tissues, cell types, physiological states, and in diseases [10,20]. To date, there is no evidence of distinct nuclear PR isoform expression linked to human endometrial function *in vivo*. Thus, one should keep in mind that the relative contribution of PRA and PRB might contribute to the diverse and indispensable actions of progesterone in cellular events in humans [24]. Furthermore, nuclear PRs exist in additional isoforms each with varying intracellular domains (Figure 1). Although PRC, PRM, and PRS have been identified in human myometrial cells *in vitro*[25,26], the essential role of these PR isoforms in the uterus *in vivo* is unknown.

Progesterone also acts through different membrane-bound PRs that mediate non-genomic activities [27] (Figure 2). Although the membrane-bound PRs appear to be expressed and regulated in normal human endometrium in a cycle-dependent manner [28], the precise mechanisms of each membrane PR in the endometrial cells remains to be determined.

In addition to the genomic and non-genomic effects of progesterone, progesterone-independent mechanisms of nuclear PR activation have been demonstrated in mouse uterine tissues [29].

### Comparison of nuclear PRA and PRB between normal and PCOS endometria

**Figure 3:**
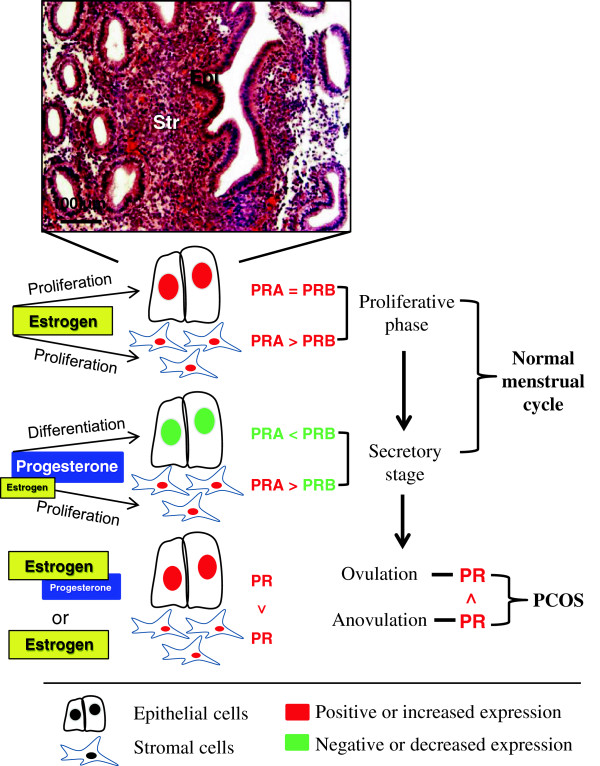
**Summary of progesterone receptor isoform expression and regulation in normal and PCOS women.** PRA and PRB are both expressed in the epithelial and stromal cells of the human endometrium, and the levels of PRA and PRB expression fluctuate in the cycling endometrium in an isoform-specific and cell-specific manner. Several studies have shown that the endometrial PR expression is altered in women with PCOS. See text for details. The normal endometrium is stained with hematoxylin and eosin. PR, progesterone receptor; Epi, epithelial cells; Str, stromal cells. See text for details.

PRA and PRB are both expressed in the epithelial and stromal cells of the human endometrium [13], and the levels of PRA and PRB expression fluctuate in the cycling endometrium in an isoform-specific and cell-specific manner [30,31] (Figure 3). During the proliferative phase, PRA expression increases at the same rate as PRB in epithelial cells but PRA expression is greater than PRB expression in stromal cells [30,32]. During the secretory phase, however, PRA expression is decreased in epithelial cells [30,31] but there is only minimal or no change in PRA expression in stromal cells [30,32] (Figure 3). Furthermore, the level of PRB expression is decreased in both epithelial and stromal cells in the secretory phase [30,31]. These data suggest that there are different sensitivities of PRA and PRB to the effects of progesterone in stromal cells. As a result of nuclear PRA- and PRB-specific functional differences [4], it is reasonable to speculate that the way in which human endometrial cells respond to progesterone *in vivo* might be determined by changes not only in the expression of the individual nuclear PR isoforms but also by ratio of PRA to PRB.

It has been reported that endometrial responsiveness to progesterone is reduced in women with PCOS [15,16], and a previous study has shown that total endometrial PR expression is higher in women with PCOS who have anovulation compared to women with PCOS who still ovulate [33]. Moreover, the increased PR expression in epithelial cells is greater than that in stromal cells in women with PCOS [34] suggesting that lower binding of progesterone in stromal cells could lead to the promotion of E2-induced epithelial cell proliferation in women with PCOS. The *in vivo* manipulation of the functional consequence of individual PR isoforms under physiological and pathological conditions in human endometrium is difficult if not impossible. However, because the PR-mediated responses depend on the coordinated, opposing, and compensatory functions of PRA and PRB [20,21], whether one isoform is more important than the other in PCOS women with progesterone resistance requires further study.

### PR-mediated signaling in endometrial progesterone resistance: the role of stromal cells

*In vitro* animal studies indicate that functional stromal cells are required for epithelial cell proliferation and differentiation in the uterus [12]. It is unclear, however, whether human endometrium has a similar paracrine regulatory interaction as seen in the uterine endometrium in animal models. PRB has been postulated to function as an anti-proliferative regulator in stromal cells, and the knockdown of PRB is reported to increase the proliferation of human endometrial stromal cells *in vitro*[35]. Interestingly, an increase in total PR expression [34] is associated with decreased expression of ki-67 (a nuclear protein that is necessary for cellular proliferation) in endometrial stromal cells in women with PCOS [19]. Although the effect of up-regulation of specific PR isoforms in women with PCOS is not clear (Figure 3), it is reasonable to hypothesize that lack of P4-induced, PR-mediated stromal cell proliferation could be a cause of progesterone resistance in women with PCOS. In addition to progesterone, nuclear PR isoforms have been shown to be under estrogenic control in human and animal uteri [29]. E2 is equally important as progesterone in females, and activation of the two estrogen receptors (ERα and ERβ) plays a pivotal role in the regulation of uterine function under both physiological and pathological conditions [29,36]. *In vivo* and *in vitro* animal studies [11] have shown that ERβ is the dominant ER in uterine stromal cells [37] and that ERβ is necessary to inhibit E2-induced epithelial cell proliferation [12]. These results suggest that stromal PR and ERβ have the same inhibitory effect on epithelial cell proliferation.

The endometrium from PCOS women with progesterone resistance often displays endometrial hyper-proliferation [8]. PRB has been shown to physically associate with ERβ in rat uterine stromal cells [38], and it has been shown that treatment with E2 induces PRB expression more than PRA expression in human endometrium [39]. Although increased PR expression [34] and decreased ERβ expression are seen in women with PCOS compared to controls [40], how epithelial cell proliferation is regulated *in vivo* by the coordinated interaction of stromal PR and ERβ in women with PCOS has yet to be determined.

Although the individual roles of PRA and PRB in the uterus have been confirmed by knockout mouse studies [22], it is not completely understood how PR-mediated gene expression in epithelial and stromal cells is regulated in the human endometrium under pathological conditions *in vivo*. However, differential changes in gene expression in the endometrium have been shown to be associated with the onset of PCOS in women [19] with progesterone resistance [16]. One intriguing finding is that the alterations in many PR-related genes are evident in women with endometriosis and accompanying progesterone resistance [41], but there are not many genes changed in women with PCOS and accompanying progesterone resistance [16]. Even though the down-regulation of PR-related mitogen-inducible gene 6 is observed in both endometriosis and PCOS [16,41], different levels of regulation of PR-related transforming growth factor β-1 is found between women with PCOS [19] and those with endometriosis [41]. Although there is conflicting reports regarding the PR-regulated MUC1expression in women with PCOS and uterine receptivity [33,34], we suspect that the different PR-related gene expression profiles between women with PCOS [33,34] and endometriosis [42] could be related to differences in PR isoform expression.

### Clinical perspectives

Treatment with progesterone appears to reverse endometrial hyperplasia in women [43], and PR-knockout mice treated with E2 develop severe uterine hyperplasia [22]. Endocrine and metabolic abnormalities that are left untreated in women with PCOS often develop into atypical endometrial hyperplasia and endometrial dysfunction-induced infertility [8]. Although progesterone-based oral contraceptive therapy is often efficacious [44], approximately 30% of women with PCOS fail to respond to such treatment [45] and progress to the development of atypical hyperplasia and further transformation to endometrial cancer. Recently, animal studies demonstrating the stromal PR-dependent antitumor effects provide a mechanistic link between progesterone action and endometrial dysfunction, in particular the development of endometrial carcinoma [46]. Therefore, loss of stromal progesterone-PR signaling in the endometrium in women with PCOS might be one of causal factors in the development of atypical endometrial hyperplasia and endometrial carcinoma [12,15].

In addition, women with PCOS often display both insulin resistance and reduced insulin responsiveness. Our laboratory and others have previously shown that the combination of metformin (*N,N*-dimethylbiguanide) and oral contraceptives are sufficient to reverse atypical endometrial hyperplasia in women with PCOS and to reverse insulin resistance in order to preserve their fertility [15,47]. Recently, we reported that the combined treatment with metformin and oral contraceptives are capable of reverting early endometrial carcinoma to normal endometria in addition to improving hyperandrogenemia and insulin resistance in PCOS women with progesterone resistance (Li et al., in submission). An important unanswered question is what links progesterone-PR signaling to atypical endometrial hyperplasia in PCOS women with progesterone resistance.

## Conclusion

The elevated estrogen and progesterone levels in women during the luteal phase of the menstrual cycle and during normal pregnancy are associated with reduced insulin sensitivity [48]. These findings suggest a potential link between the activation of steroid receptor signaling and insulin resistance. Combined treatment with metformin and Diane-35 might provide the beneficial effects on progesterone and insulin resistance as well as reverting early endometrial carcinoma, but knowledge about how their co-effects are exerted is limited. We note that the absence of progesterone action or an imbalance between E2 and progesterone allows E2 to induce oncogenesis [12], and progesterone resistance is associated with insulin resistance and dysregulation of endometrial stromal PR activity [45]. It has been reported that either one or both of PRA and PRB expression is decreased in women with endometrial carcinoma compared to those with normal endometrium [49]. Moreover, treatment with metformin not only inhibits P450 aromatase activation and suppresses endometrial cell proliferation [50], but it also reduce progesterone resistance and increases PR expression in endometrial cancer cells [51,52]. Therefore, a possible scenario is that endometrial cells coexpress receptors for insulin/insulin-like growth factor-1 (IGF-1) [53-56] and progesterone/E2 [31,57] and that metformin and Diane-35 coordinate the regulation of insulin/IGF-1 receptor, PR, and ER levels. This would result in a significant increase in the number of receptor molecules available for signaling in the endometrium in PCOS women with progesterone resistance. How a positive effect of metformin combined with Diane-35 at the molecular level could inhibit the development of atypical endometrial hyperplasia and endometrial carcinoma is a matter for further investigation. We believe that identifying undiscovered mechanistic pathways in the endometrium from PCOS women with both progesterone and insulin resistance remains an attractive research approach to understanding the pathogenesis of atypical endometrial hyperplasia and endometrial carcinoma.

## Competing interests

The authors have nothing to disclose.

## Authors’ contributions

XL and RS wrote and edited the manuscript. JFL, HB, and RS provided conceptual input. YF and RS assembled the figures. All authors participated in the discussion and approved the final submitted version.
